# Borate of Soda in Dysmenorrhœa

**Published:** 1837

**Authors:** Daniel Sthal

**Affiliations:** Vincennes, Indiana


### Art. V.—Borate of Soda in Dysmenorrhœa.

*Cases showing the efficacy of Borax in Dysmenorrhœa.* By Daniel Sthal, M. D., of Vincennes, Indiana.

*Dysmenorrhœa* is one of those diseases with which we have often to deal, and with which we are the least acquainted. Nothing can be more disagreeable to a physician, than to treat a case of whose nature i. e., pathology, he is ignorant: we discriminate the case by its symptoms, we give our drugs, and why? because doctor such an one has prescribed them with success, or professor such an one has recommended them. We give them therefore, and may succeed; but no one will be satisfied in his mind with such empiricism, unless he is one of the kind of "doctors" who are always ready to swear in verba magistri, and who have a ready prescription for every disease. But disagreeable as it may be, to give medicines without knowing the "why's," and "wherefore's," still we are very often under the necessity of doing so. There are diseases, whose nature is as yet a mystery to us, and I am afraid will be, as long as nature persists in keeping the curtain before her laboratory. If it is, therefore, beyond the reach of the human mind to ascertain the pathology or *causa proxima* of such cases, we should nevertheless, endeavor to find out the means of cure, which we can only do by means of experiment; and it is the imperious duty of every practitioner, to impart his experience successful or unsuccessful, to the medical world; and by that means he at least benefits the practical part of our profession directly. We do not know, for instance, the pathology of intermittent fever; perhaps we never shall know it, but we are, by long experience, pretty *sure* to prevent a second attack of a tertian ague, if we make a liberal use of the cinchona or its preparations.

This I advance as an apology for writing on a subject handled so often, and so ably by older and more experienced members of the profession. But the discussions on this subject are very far from being closed; a successful plan of treatment has not yet been established, and as my experience in the treatment of the disease in question is any thing but discouraging as to the final cure thereof, I could not withhold any longer this communication. Should it prove successful, I shall find my reward in the consciousness of having done something for my profession; in the contrary case, I wish this paper may share the fate of so many others, i. e., be read and forgotten.

Obstinate cases of dysmenorrhœa are a great source of trouble to a physician, especially to the juniors in the profession; who, applying the remedies recommended by standard authors, and disappointed in their anticipations—mistrust at last their books and their own judgment. Thus it was with me, until I discovered the effect of borax in this disease, and with great satisfaction, founded upon experience in a vast number of cases, I can recommend it as *a sure and safe remedy in dysmenorrhœa*, and will only add, that it has been tried by some of my medical friends here, with the same gratifying result. As to its modus operandi I know nothing, and leave it to the reader to conjecture ad libitum about it.

If we would expect a salutary effect from borax in dysmenorrhœa, we must not give it indiscriminately in every case, and not without preparing the system for its reception. In lymphatic constitutions and with patients who are debilitated by other diseases, the effect is often doubtful, at least not very prompt; whereas in patients of sanguineous or sanguineo-nervous constitutions, and where there is an actual plethora, it will always be found effectual if venesection be premised. We should never give it without bringing all the systems of the organism to a kind of equilibrium; therefore bleed and give antiphlogistics in plethoric constitutions, raise the energy of the nervous system in "nervous subjects," and give tonics to the debilitated.

After many trials with borax, I determined on the following plan of treatment, and follow it now exclusively.

In plethoric habits,* I take from four to six ounces of blood from the veins of one of the lower extremities, and repeat the V. S. if necessary, and keep the bowels open by epsom salts. For two days previous to the appearance of the menses, I order a warm foot bath every evening and morning, and give borax in the following formula

℞. Natri. borac, ʒii.

Infusi semini lini. ℥vii. Misce.

Every two hours a table spoonfull to be taken.

The pain I relieve by exractum hyoscyami† one grain every 15 or 30 minutes.

In nervous habits where I find this complaint in four cases out of seven, I commence my treatment with the exhibition of tinctura valerianæ teaspoonfull doses thrice a day, and in days previous to the appearance of the menses, I give borax, etc.; as above.

As soon as the menstruation ceases for the time, I treat plethoric habits with slight antiphlogistics and with the nervous, I resume the use of tinctura valerianæ until a day or two previous to menstruation, when I act as before. After the second appearance of the menses, after following that plan, I will generally hear the patient relate with gratification, that the pain was less and of shorter duration, that she menstruated more than usual, and that she feels more contented and cheerful,‡ and by the third time, the complaint, the mere idea of which haunted the poor woman from one period to the other, is generally removed.

Particular attention is to be paid to the *diet* of the patient. She is only to be allowed light and easy digestible food, as chicken, squirrel, veal, light bread, tea, etc. Coffee, spirits, fat meat, cheese, saurkraut, pork, etc., are to be prohibited.

Before I relate some cases in order to illustrate the foregoing plan of treatment, I must remark, that as I noticed them only

---

*It is very rare to find dysmenorrhœa in plethoric habits.

† In some cases the extr. hyoscyami produced nausea, and if there was no contraindication I gave Dover's powder or what was the most effectual—Aqua Laurocerasi.

‡In most cases of dysmenorrhœa menstruation is scanty, and symptoms of hysteria are present.

cursorily in my case-book, for my own information, I am not able to give the history of the cases minutely and extensively.

*Case* 1. Mrs. T. of York county, Pennsylvania, about 26 æt.; sanguineo-nervous temperament, light hair and florid complexion, suffered for a number of years, with dysmenorrhœa. She had been married about three years, at the time when I first saw her, in 1833, but had never been pregnant. She menstruated regularly every four weeks, but her sufferings where then so severe, that she left no remedy untried in hopes of relief from torments which embittered her life, and which could be ameliorated neither by her own strength of mind, nor by the soothing endearments of husband or friends. During the intervals of menstruation, she was in relative good health. She had been attended by good physicians and by quacks, and the catalogue of all the remedies usually applied in such cases, was exhausted in vain. Having just arrived from Germany, she at once fancied, that I was sent by providence to relieve her sufferings, and she applied immediately to me. I prescribed all the remedies recommended by my teachers and in books, but without the least good effect. Finding the energy of her nervous system rather impaired, while the actions thereof were increased, I gave her the tinctura valerianæ, etc., as above mentioned; and having now learned that her menses were scanty, and being aware of the emmenagogue property of borax venetus, I administered it in the above mentioned form, and to my great satisfaction, I was informed that her menstruation took place with less pain. I now continued the treatment. The next time she was perfectly easy, and the time following, no menstruation took place, nature being enabled to pay the tribute which she owes to matrimonial life.

This case encouraged me to further trials, and the result was equally gratifying. But as the cases were less remarkable, and as I had at the time very little leisure to record them, I shall only point out a few more, of recent date.

Mrs. W., of Sullivan, co., I. about 30 æt., the mother of four children, of which the youngest was 4 years old. She is of a sanguineo-nervou s temperament, had been in delicate health for some time back, which was principally attributed to hysteria, she has always regularly menstruated until since she weaned the last child. She was afflicted with dysmenorrhœa with the most agonizing pains. Her menstruation was profuse. As she was very weakly and the sensibility much increased, while the energy of the nerves was impaired, I gave January 5th, 1836, the tinct. valerianæ, for about three weeks, then gave the borax and during the pains extr. hyoscyam. as above mentioned. At the next menstruation (January 30th,) the pain was considerably less and shorter in duration. I now continued the same treatment, and in the course of two months after that, I had the satisfaction to hear her say, that she was as well now as ever she was in her life, her menstruation being without pain and regular, and she still continues so.

Case 3. Miss B., 17 æt. of sanguineo lymphatic habitus, had been menstruating for about three years; but always with much pain, at irregular times and scanty. As there was no indication for active depletion, and as there were symptoms of gastric irritation, I gave her a dose of magnesiæ sulphas, and treated her after that, with slight antiphlogistics, ordered pediluvia, and about a day before she thought her menses would appear, I gave her borax in the above mentioned dose and form, and shortly after her menstruation had taken place, she told me that she menstruated easier, and in greater quantity, she was however very weak now, was dejected in mind, had occasionally nervous headache, etc., and this prompted me to abandon the antiphlogistic treatment, and to give her the tinct. valerianæ. The next menstruation was with less pain still, her general health improved, and she took no medicine any more. I saw her father some two months' after she had taken the last medicine, and he told me that she was quite well and in good spirits.

*February 1st,* 1837.

